# The effect of quercetin on obesity and reproduction through the expression of genes involved in the hypothalamus-pituitary-gonadal axis

**DOI:** 10.5935/1518-0557.20240097

**Published:** 2025

**Authors:** Elaheh Shams, Dina Zohrabi, Ozra Omrani, Mohammad Hossein Sanati, Maryam Karimi-Dehkordi, Nasrin Yazdanpanahi, Fatemeh Khademi Moghadam, Vahid Zarezade

**Affiliations:** 1 Behbahan Faculty of Medical Sciences, Behbahan, Iran; 2 Higher Education Institute, Meymeh, Iran; 3 Department of Medical Genetics, National Institute of Genetic Engineering and Biotechnology, Tehran, Iran; 4 Department of Clinical Sciences, Faculty of Veterinary Medicine, Shahrekord Branch, Islamic Azad University, Shahrekord, Iran; 5 Department of Biochemistry, Falavarjan Branch, Islamic Azad University, Isfahan, Iran; 6 Department of Biology, Faculty of Science, Shahid Chamran University of Ahvaz, Ahvaz, Iran

**Keywords:** Quercetin, Obesity, Ovarian Follicle, Gene expression, Reproduction

## Abstract

**Objective:**

Quercetin is a flavonoid compound extracted from fruits and plants and is used as a natural antioxidant to prevent or treat a variety of diseases such as cancer, obesity, chronic inflammation, and reproductive system dysfunction. The aim of this study was to investigate the effects of quercetin on obesity and ovarian tissue by analyzing the expression of genes involved in the hypothalamus-pituitary-gonadal axis, including *ob-Rb, ob-Ra*, and brain-derived neurotrophic factor (*Bdnf*), neuropeptide Y (*NPY*), and Kisspeptin (*Kiss-1*).

**Methods:**

In this experimental study, female rats were divided into three groups, and the effect of quercetin with doses of 50 and 100 mg/kg on weight and BMI was investigated. Also, the gene expression was assessed using the real-time PCR technique. The estrogen, progesterone, FSH, and LH were assessed using the chemiluminescence technique. The diameter and number of different types of follicles, corpus luteum, and blood vessels in mice were investigated. The growth indicators of the children, including the number, weight, and height and head width of the born children, were checked.

**Results:**

Quercetin caused a decrease in BMI, a significant increase in the expression of *ob-Rb, ob-Ra*, and *Bdnf* genes, a significant decrease in the expression of *NPY* and *Kiss-1* genes, and led to an increase in sex hormones. Quercetin improved follicular indices and increased the number of mouse embryos.

**Conclusions:**

Probably, quercetin affects the hypothalamus-pituitary-gonadal axis by changing the expression of genes, and it helps to reduce obesity and increase fertility and better function of the reproductive system.

## INTRODUCTION

**Figure 1:**
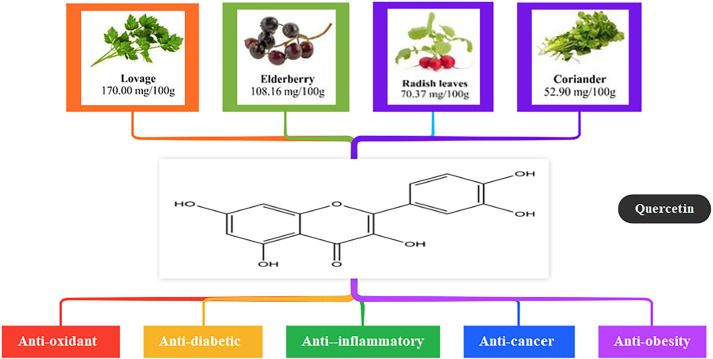
Food sources and contents of natural quercetin and its biological effects.

Quercetin (2-(3,4-dihydroxy phenyl)-3,5,7-trihydroxy-4H-1-benzopyran-4-one) (Figure 1) is a kind of natural flavonoid found in large quantities in fruits and vegetables and has a wide range of physiological effects, including antioxidant, antidiabetic, anti-inflammatory, and anticancer functions. Quercetin is a natural compound for the treatment of reproductive disorders (Zheng *et al.,* 2022). In addition, quercetin was effective in reducing visceral fat in adipose tissue (Michala & Pritsa, 2022).

Body fat affects hypothalamic-pituitary-gonadal (HPG) axis function in women (Salem, 2021). Obesity causes reproductive disorders by changing the expression of genes (Shirvanizadeh *et al.*, 2023). Obesity is associated with anovulation, infertility, abortion, menstrual disorders, uterine bleeding, preeclamsia, gestational diabetes, and adverse results in assisted reproduction methods (García-Ferreyra *et al.*, 2021). Adipose tissue may influence ovarian and endometrial function through many factors, such as leptin, free fatty acids, and cytokines (Gambineri *et al.,* 2019).

Leptin is a peptide hormone and is derived from the mRNA transcript of the *ob* gene. Mice have six binding isoforms of the leptin receptor (ob-Ra to ob-Rf). Leptin is secreted by adipocyte (fat) cells and plays important roles in regulating food intake, energy consumption, body weight through the central nervous system, regulation of body temperature, and balancing kidney metabolisms by targeting the hypothalamus (Childs *et al*., 2021). In recent years, leptin showed stimulatory effects on gonadotropin secretions in some mammalian species. Leptin receptor activation triggers the STAT3 signaling pathway, which is responsible for the LH surge (Zeng *et al*., 2018).

Brain-derived neurotrophic factor (Bdnf) and leptin work well together. Bdnf is a member of the neurotrophin family that has been linked to metabolic effects such as fat and sugar metabolism and plays a critical function in the reproductive process of female animals. Bdnf levels have been shown to be low in patients with weak ovaries (Chow *et al*., 2020). Neuropeptide Y (NPY) is an appetitive agent with a potent central effect that is down-regulated by leptin. The present findings suggest that NPY may serve as a neurobiological brake on the sleep of kisspeptin neurons (Bano *et al.,* 2022). Kisspeptin is a peptide encoded by the *Kiss-1* gene. This peptide is essential for regulating hypothalamic-pituitary-gonadal axis activity (Padda *et al.,* 2021).

Quercetin has played an effective role in improving the symptoms of polycystic ovary disease and ovarian cancer, and researchers suggest that it can be an ideal agent or an auxiliary agent for the treatment of cancer and other diseases (Shafabakhsh & Asemi, 2019). Considering the various medicinal properties of quercetin, this study was conducted to investigate the phytochemical role of this compound on ovarian tissue and to investigate the effects of quercetin on the hypothalamus-pituitary-gonadal axis in healthy female rats.

## MATERIALS AND METHODS

### Animals

All animals were kept in standard cages with adequate food (prepared and special pellets) and water (tap water) at 23±2°C in 12 hours of light and 12 hours of darkness. In this experiment, adult female rats (200 g±5 g) were used. To evaluate the effect of the drug, all rats underwent a sexual cycle. For this purpose, progesterone (Toliddaru Company, IRAN) was injected once with a dose of 50 micrograms, and 24 hours later, estradiol valerate (Toliddaru Company, IRAN) at a dose of 100 micrograms was dissolved in 2 cc of olive oil. After 6 hours, a smear was obtained from the vagina, and the rats in the same cycle were selected. After that, the mice were randomly divided into 3 groups of 6 (Group 1: The group that received only tween 80 solution as quercetin (Sigma, Germany) solvent). Group 2: received quercetin at a dose of 50 mg/kg. Group 3: received quercetin at a dose of 100 mg/kg). Intraperitoneal injections were given every two days for thirty days. The weight of the mice on the first, fifteenth, and thirty days of the experiment using a laboratory weighing scale (EK-model 4000H, AND company, America) was measured and recorded with an accuracy of 0.1, and their weight was measured in millimeters at the distance from the mouth to the anus with a metal ruler, and the body mass index (BMI) value of each was checked.

### Assessment of gonadotropin hormones

After injection of treatments into all groups, rats were anesthetized with 100 µl of ketamine and xylazine in a ratio of 70 to 30. 4 to 5 cc of blood was collected from the left ventricle of the heart with a syringe, and the serums were separated. An electrochemiluminescence immunoassay (DxI800 automated chemiluminescence assay and commercial kit; Beckman Coulter, Inc., CA, USA) was used to determine hormone levels.

### Histological investigation

The left ovary of mice was fixed in a 10% formalin solution. Then it was paraffinized and cut into 3-4 µm sections. Then these sections were stained with hematoxylin and eosin, and then ovarian follicles were counted using a light microscope (Olympus IX71, Japan).

### RNA extraction and real-time PCR

The hypothalamus was extracted from each rats’ brain area and utilized to measure gene expression levels. The RNA extraction from the hypothalamus gland was carried out using the QIAGEN RNeasy Plus mini kit according to the manufacturer’s instructions, and the extracted RNA amount was measured using a nanodrop (Nanodrop Technology, Wilmington, USA). The QIAGEN cDNA synthesis kit methodology was used while synthesizing cDNA. The cDNA was produced and kept at -70°C until used. Primer Premier 5 software (Premier Bio Soft International, Palo Alto, CA, USA) was used to generate primers for the following genes: ob-Ra, ob-Rb, NPY, Bdnf, Kiss-1, and β-actin (housekeeping gene). A Rotor-Gene 6000 system (Corbett Life Science, Mortlake, Australia) with the SYBR green master mix kit (Parstous, Iran) was used to produce real-time PCR experiments (Table 1).

**Table 1:** Sequence and specifications of primers.

Primer	Sequence	PCR product length (bp)
*ob-Rb*	5' CTGTGTAGTGTGAGGAGG 3' 5’ AAGGGAGGCACCGATGG 3’	109
*ob-Ra*	5' CTCTTGTGTCCTGCTGC 3' 5’ GACTGTTGGGAGGTTGG 3’	107
*NPY*	5' CCCAGAGCAGAGCACC 3' 5’ AGCAGGGATAGAGCGAG 3’	104
*Bdnf*	5' CCCTTCTACACTTTACCTC 3' 5’ TTCACCCTTTCCACTCCT 3’	202
*Kiss-1*	5' TGCTGCTTCTCCTCTGTG 3' 5’ ACGAGTTCCTGGGGTCC 3’	106
*β-actin*	5' CCATCTATGAGGGTTACGC 3' 5’ TGTAGCCACGCTCGGTC 3’	104

### Evaluation of number and growth indices (weight, height and head width) of children born in control groups with experimental groups

At this stage, rats in 3 groups of 4 (in each group, male rats and 3 female rats) were treated in the same way as before. Then mating was performed between rats naturally so that female rats became pregnant. Female rats were used that did not mate before the test. In late pregnancy, pregnant animals were placed in separate cages. After the birth of children in the study groups, their number was counted, and growth indices (weight, height, and head width) were measured on the fifth, ninth, fifteenth, twenty-third (time of weaning children), and thirtieth days. The measuring instrument included a digital scale to determine the weight, a vernier caliper with an accuracy of 0.01 to measure the width of the head, and a ruler to measure height.

### Statistical analysis

The obtained results were statistically analyzed by SPSS 19.0 software. A one-way ANOVA test was used to examine the differences and the significance of the data, and *p*<0.05 was considered an acceptable criterion for significant differences. Then the graphs were drawn by Excel software. Prism software was also used.

## RESULTS

### The effect of quercetin on the weight of rats

The average weight of the examined mice was 200 grams; in the control group, the weight of the mice increased on the 30th day (205.2±2.005), and in the group treated with quercetin, the weight of the mice decreased. The highest weight loss was observed in the group treated with quercetin 50 mg/kg dose (181.2±0.5575) (Table 2).

**Table 2:** Effect of quercetin on weight and BMI.

Variable	Growth stage	Control group (Tween)	Quercetin 50 mg/kg	Quercetin 100 mg/kg
		Mean±SD	Mean±SD	Mean±SD
**weigh (g)**	Day 1	200.5±1.588	199.7±1.621	199.5±1.674
	Day 15	202.5±1.680^*^	190.4±0.9326^***^	193.9±1.971^***^
	Day 30	205.2±2.005^***^	181.2±0.5575^***^	189.4±2.313^***^
**BMI**	Day 1	0.5700±0.02301	0.5667±0.02029	0.5639±0.01819
	Day 15	0.5900±0.008402	0.5550±0.02358	0.5500±0.03804
	Day 30	0.6033±0.03343^**^	0.5350±0.03400^**^	0.5433±0.01283

### The effect of quercetin on the expression of genes

**Figure 2:**
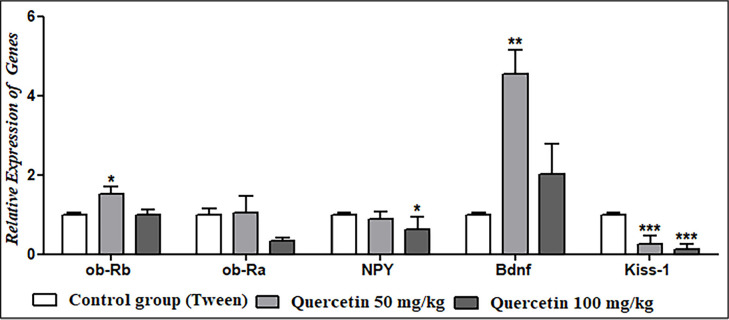
The effect of quercetin on the expression of genes compared to the tween control group.

The results of real-time PCR revealed that a dose of 50 mg/kg of quercetin caused a significant increase in the expression of the *ob-Rb* and *ob-Ra* genes; this increase was significant for the *ob-Rb* gene, but a dose of 100 mg/kg of quercetin did not alter the expression of this gene and decreased the expression of the *ob-Ra* gene. Moreover, both 50 and 100 mg/kg of quercetin significantly decreased the *NPY* and *Kiss-1* gene expression, and both 50 and 100 mg/kg of quercetin significantly increased the expression of the *Bdnf* gene (Figure 2).

### The results of the study of hormones

The results indicated that the levels of estrogen, progesterone, FSH, and LH increased in healthy rats receiving quercetin compared to the control group, which was significant for FSH at both doses (*p*<0.001) and for estrogen in the group quercetin 100 mg/kg (*p*<0.05) (Table 3).

**Table 3:** Effect of quercetin on serum estrogen, progesterone, FSH and LH levels.

Variable name	Control group (Tween)	Quercetin 50 mg/kg	Quercetin 100 mg/kg
**Estrogen**	32.55±1.8	32.63±0.64	35.2±2.3^*^
**progesterone**	2.04±0.09	2.09±0.015	2.06±0.01
**FSH**	0.33±0.03	0.37±0.03^**^	0.36±0.01^**^
**LH**	0.56±0.01	0.60±0.02	0.63±0.01

* Shows a significant difference compared to the tween control group at the level of *p*<0.05

** Shows a significant difference compared to the tween control group at the level of *p*<0.001

### Ovarian morphometric results

#### Results of experiments on the number of follicles, corpus luteum and number of blood vessels

**Figure 3:**
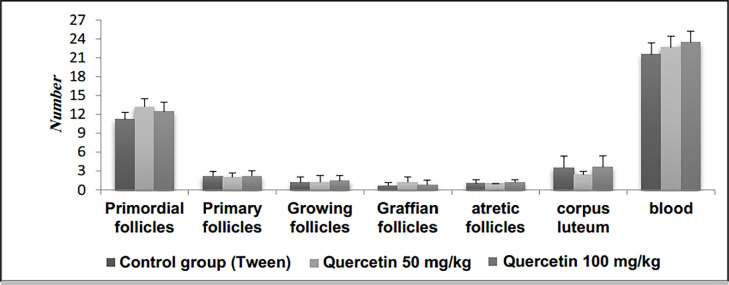
The number of follicles, corpus luteum and number of blood vessels in groups receiving quercetin 50 and 100 mg/kg in comparison to the tween control group.

The average number of follicles, corpus luteum, and blood vessels in the groups treated with quercetin increased compared to the control group, but there was no significant change (Figure 3).

#### Experimental results on follicle diameter and corpus luteum diameter

**Figure 4:**
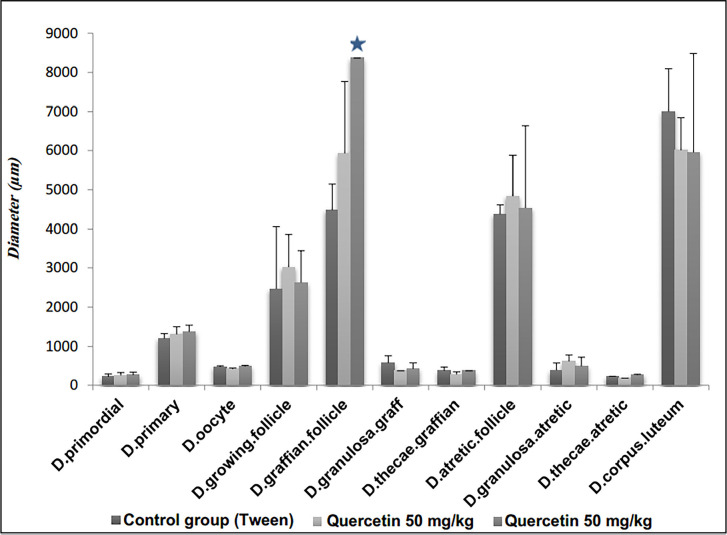
The effect of quercetin 50 and 100 mg/kg on follicles and corpus luteum diameter in comparison to the tween control group.

**Figure 5:**
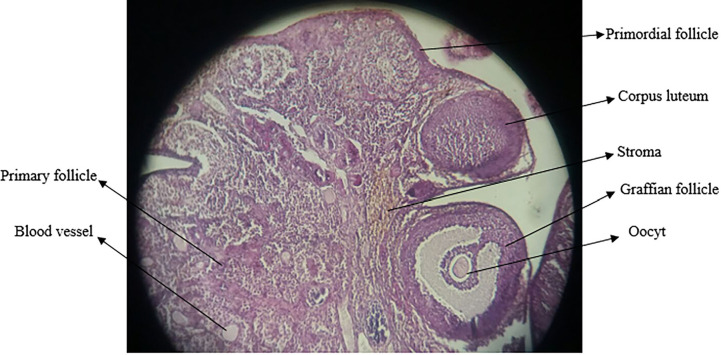
Photomicrograph of ovarian tissue.

The mean diameter of follicles and corpus luteum in the quercetin-treated groups did not change significantly compared to the control group; only the diameter of the follicle graph in the quercetin-receiving group at a dose of 100 mg/kg showed a significant increase (*p*<0/05) (Figure 4). The photomicrograph of ovarian tissue is shown in Figure 5.

#### Results of experiments on the number of children and growth indicators

Quercetin led to a considerable rise in the number of children. The weight of newborns on the ninth and fifteenth days in the group receiving quercetin at a dose of 50 mg/kg, as well as on the twenty-third and thirtyth days in both doses, declined considerably. Neonatal head width did not change in the experimental groups, but the height index of the babies in the experimental groups showed a significant decrease compared to the control group (Table 4).

**Table 4:** Descriptive and inferential statistics related to the effect of quercetin on the number of children and growth indices.

Variable	Growth stage	Control group (Tween)		Quercetin 50 mg/kg		Quercetin 100 mg/kg	
		N	Mean±SD	N	Mean±SD	N	Mean±SD
**weight (g)**	Day 5	20	8.87±0.87	30	8.24±0.57	22	8.66±1.29
	Day 9	19	14.94±1.3	27	13.47±0.78^*^	21	13.11±1.9^*^
	Day 15	18	22.73±1.2	27	19.88±0.88^**^	21	22.5±1.26
	Day 23	17	39.24±2.7	25	35.73±0.64^***^	20	29.4±1.78^***^
	Day 30	17	69.45±4.6	25	51.28±3.97^***^	20	41.81±2.87^***^
**head width (cm)**	Day 5	20	1.23±0.08	30	1.21±0.09	22	1.22±0.07
	Day 9	19	1.46±0.08	27	1.4±0.08	21	1.4±0.07
	Day 15	18	1.61±0.08	27	1.6±0.08	21	1.62±0.07
	Day 23	17	1.82±0.4	25	1.8±0.5	20	1.83±0.4
	Day 30	17	2.3±0.21	25	2.2±0.26	20	2.2±0.20
**height index (cm)**	Day 5	20	8.3±0.74	30	8±0.23	22	8.21±0.54
	Day 9	19	10.69±0.44	27	9.98±0.26^***^	21	9.52±0.44^***^
	Day 15	18	13.78±0.35	27	12.65±0.35^***^	21	12.67±0.39^***^
	Day 23	17	17.91±0.67	25	17.57±0.11^*^	20	15.89±0.59^***^
	Day 30	17	23.5±0.81	25	21.15±0.63^***^	20	19.87±0.68^***^

* Shows a significant difference compared to the control group at the level of *p*<0.05

** Shows a significant difference compared to the control group at the level of *p*<0.01

*** Shows a significant difference compared to the control group at the level of *p*<0.001

## DISCUSSION

Natural compounds are the most important reservoirs of antioxidants (Shams *et al.*, 2024a). Quercetin is a plant flavanol that has antioxidant, anti-inflammatory and anti-obesity effects (Ezzati *et al.,* 2020; Sun *et al*., 2024). Gnoni *et al*. (2022) reported quercetin significantly reduces the expression of SREBP-1 and XBP-1 genes, and their lipogenic gene targets, and has a direct anti-lipogenic effect. There is a close relationship between obesity and reproductive system function in women. The study of Soleimani Mehranjani *et al*. (2021) has shown that quercetin can improve the tissue quality and function of mouse ovaries. Quercetin has been found to have genomic actions regulating the expression of several genes (Ghafouri-Fard *et al.,* 2021).

Our results showed that the weight of mice in the groups treated with quercetin 50 and 100 mg/kg was reduced compared to the control group, and this weight loss was significant in the 50 mg/kg dose. Also, quercetin at a dose of 50 mg/kg increased the expression of *ob-Ra* and *ob-Rb* genes, and at both doses of 50 and 100 mg/kg, the gene expression of *Bdnf* was increased. Leptin and *Bdnf* pathways are important players in body weight homeostasis and reproduction (Scabia *et al.,* 2018). After food intake, leptin is produced, which sends a signal to the hypothalamus that inhibits the appetite (Wróbel-Biedrawa *et al.,* 2022). Numakawa *et al*. (2014) study showed that hippocampal *Bdnf* expression and its receptor “TrkB” were decreased in leptin-deficient mice with obesity, which were improved by adrenalectomy or low-dose corticosterone replacement. The study of Rahvar *et al*. (2018) showed that quercetin at doses of 20 and 50 mg/kg caused a significant increase in the mRNA expression of *Bdnf* as compared with the control group. In a similar work, Pazoki & Arshadi (2019) reported that 8-week consumption of flax seed increased *Bdnf* and a decrease in IGF-1 levels and then reduced the body weight and improved metabolic parameters in obese women. Neuropeptide Y neurons are one of the main targets for leptin that can increase appetite and reduce energy consumption, consequently causing the accumulation of fat in the body and leading to weight gain and obesity (Oztas *et al.,* 2021). Thus, leptin can decrease obesity by inhibiting the neuropeptide Y and increasing energy expenditure and metabolism levels (Babaei *et al*., 2017). The results of current work indicated that quercetin in both 50 and 100 mg/kg decreased the *NPY* gene expression. There is a complex interplay between neuropeptide Y and kisspeptin. Since the last two decades, the dynamic role of kisspeptin in regulating the reproductive axis has been well characterized (Terasawa & Garcia, 2020). Direct inhibition of kisspeptin neurons by neuropeptide Y highlights the importance of these two neuropeptides in the control of reproductive axis activity (Bano *et al.,* 2022). In the present study, *KISS-1* gene expression was significantly decreased in doses of 50 and 100 mg/kg quercetin. Zhang *et al.* (2022) study has shown that quercetin leads to a decrease in *KISS-1* gene expression.

Every gene covered above is essential to reproduction. It seems that the health of the ovarian follicles and their capacity to generate additional hormones like progesterone and LH may be directly assessed by measuring their leptin levels (Misch & Puthanveetil, 2022). The study conducted by Farhadi *et al*. (2022) has established a correlation between the rise in estrogen and the rise in leptin. Leptin can increase the release of FSH and LH and functions as a metabolic signal that stimulates the reproductive system (Sengupta *et al.,* 2019). According to a study by Seo *et al*. (2018), BDNF and estrogen interact in a variety of complex ways in the central nervous system. The induction of BDNF may contribute to the effects of estrogen, at least somewhat. NPY is known to control female reproductive function via the central nervous system (Shams *et al.*, 2024b). NPY decreases GnRH/LH secretion in a variety of animals, including nonhuman mammals (Bano *et al.,* 2022). The current investigation found that healthy rats receiving quercetin had higher mean blood levels of estrogen, FSH, and LH compared to the control group. FSH levels were significantly higher at both dosages (*p*<0.001), consistent with previous studies examined. In fact, the expression of genes ob-Rb, ob-Ra, and Bdnf rose, both of which have a direct association with estrogen and the hormones LH and FSH, while the expression of gene NPY dropped, which inhibits the hormone LH, indicating HPG axis adjustment. The Bin-Jaliah (2021) study reveals that quercetin inhibits the ROS-p53-Bax-caspase-3 apoptosis axis and increases FSH, LH, and testosterone.

In the present study, the average number and diameter of follicles, corpus luteum and blood vessels in the groups treated with quercetin increased compared to the control group, but there was no significant change; only the diameter of the follicle graph in the group receiving quercetin with a dose of 100 mg/kg showed a significant increase (*p*<0.05). So, it can be concluded that quercetin has a favorable effect on the ovarian tissue and the folliculogenesis process in the doses used. Quercetin has properties similar to other flavonoid phytoestrogens that have a dose-dependent effect (Carmona-Aparicio *et al.,* 2019). In this study, the indicators of children were also studied, and a significant increase in the number of children and a decrease in the weight of babies were observed in the groups receiving quercetin. Increasing the concentration of leptin after ovulation is also directly related to increasing the ability of the fetus to implant (Gupta *et al.,* 2020). Experiments show that leptin levels are higher in blastomere inner cells, and it is likely that leptin plays a vital role in the early stages of embryonic development before implantation (Kotlarska *et al.,* 2021). Rashidi *et al*. (2021) study shows that quercetin increases the quality of eggs and embryos and reduces oxidative stress in granulosa cells, and can be used as a complementary treatment and has positive effects in the treatment, of ovarian-related.

Quercetin, in a dose-dependent manner, led to a significant increase in the expression of the *ob-Rb, ob-Ra,* and *Bdnf* genes, a slight decrease in the expression of the *NPY* gene, and a significant decrease in the expression of the *Kiss-1* gene. Quercetin also increased the amount of sex hormones and caused a slight increase in follicular indices, but it was not significant and led to an increase in the number of embryos in mice. Finally, the results showed that quercetin probably affects the hypothalamus-pituitary-gonadal axis by changing the expression of genes and using quercetin as a medicinal or food supplement can help reduce obesity and increase fertility and better functioning of the reproductive system in women.
